# Selenium Deficiency Is Widespread and Spatially Dependent in Ethiopia

**DOI:** 10.3390/nu12061565

**Published:** 2020-05-27

**Authors:** Adamu Belay, Edward J. M. Joy, Christopher Chagumaira, Dilnesaw Zerfu, E. Louise Ander, Scott D. Young, Elizabeth H. Bailey, R. Murray Lark, Martin R. Broadley, Dawd Gashu

**Affiliations:** 1 Center for Food Science and Nutrition, Addis Ababa University, Addis Ababa P. O. Box 1176, Ethiopia; adamu_bel2000@yahoo.com; 2 Food Science and Nutrition Research Directorate, Ethiopian Public Health Institute, Gulele Sub City, Addis Ababa P.O. Box 1242, Ethiopia; dilnesaw2012@gmail.com; 3 Faculty of Epidemiology and Population Health, London School of Hygiene & Tropical Medicine, Keppel Street, London WC1E 7HT, UK; edward.joy@lshtm.ac.uk; 4 School of Biosciences, University of Nottingham, Sutton Bonington Campus, Loughborough, Leicestershire LE12 5RD, UK; christopher.chagumaira@nottingham.ac.uk (C.C.); scott.young@nottingham.ac.uk (S.D.Y.); liz.bailey@nottingham.ac.uk (E.H.B.); murray.Lark@nottingham.ac.uk (R.M.L.); martin.broadley@nottingham.ac.uk (M.R.B.); 5 Department of Crop and Soil Science, Bunda Campus, Lilongwe University of Agriculture and Natural Resources (LUANAR), Lilongwe P.O. Box 219, Malawi; 6 Inorganic Geochemistry, Centre for Environmental Geochemistry, British Geological Survey, Nottingham NG12 5GG, UK; land@bgs.ac.uk

**Keywords:** serum selenium, geospatial prediction, glutathione peroxidase 3, iodothyronine deiodinase, Ethiopia

## Abstract

Selenium (Se) is an essential element for human health and livestock productivity. Globally, human Se status is highly variable, mainly due to the influence of soil types on the Se content of crops, suggesting the need to identify areas of deficiency to design targeted interventions. In sub-Saharan Africa, including Ethiopia, data on population Se status are largely unavailable, although previous studies indicated the potential for widespread Se deficiency. Serum Se concentration of a nationally representative sample of the Ethiopian population was determined, and these observed values were combined with a spatial statistical model to predict and map the Se status of populations across the country. The study used archived serum samples (*n* = 3269) from the 2015 Ethiopian National Micronutrient Survey (ENMS). The ENMS was a cross-sectional survey of young and school-age children, women and men. Serum Se concentration was measured using inductively coupled plasma mass spectrometry (ICPMS). The national median (Q1, Q3) serum Se concentration was 87.7 (56.7, 123.0) μg L^−1^. Serum Se concentration differed between regions, ranging from a median (Q1, Q3) of 54.6 (43.1, 66.3) µg L^−1^ in the Benishangul-Gumuz Region to 122.0 (105, 141) µg L^−1^ in the Southern Nations, Nationalities, and Peoples’ Region and the Afar Region. Overall, 35.5% of the population were Se deficient, defined as serum Se < 70 µg L^−1^. A geostatistical analysis showed that there was marked spatial dependence in Se status, with serum concentrations greatest among those living in North-East and Eastern Ethiopia and along the Rift Valley, while serum Se concentrations were lower among those living in North-West and Western Ethiopia. Selenium deficiency in Ethiopia is widespread, but the risk of Se deficiency is highly spatially dependent. Policies to enhance Se nutrition should target populations in North-West and Western Ethiopia.

## 1. Introduction

Micronutrients are required in small amounts in the diet and are essential for maintaining human health. Selenium (Se) is an important trace element for human health [1,2]. Deficiency of Se mainly occurs due to inadequate intake of the nutrient from the diet. The deficiency can result in impaired expression or activity of Se-containing enzymes such as glutathione peroxidase 3 (GPx3) important for antioxidant function, and iodothyronine deiodinase (IDI) which is important for normal thyroid function. The effects of Se deficiency are wide-ranging, from impaired cognitive development, through to greater risks of non-communicable diseases [2,3,4,5].

Despite the importance of Se in human health, deficiency is widespread. However, the distribution of deficiency risks is highly variable, which poses challenges for policy makers for targeting appropriate interventions. There are few data on population Se status globally, especially in sub-Saharan Africa [6,7]. The prevalence of Se deficiency on national scales in Africa was estimated indirectly, based on Se supplies in food systems, revealing that approximately 22% of the population of Africa is at risk of Se deficiency, with the prevalence of deficiency exceeding 60% in eight countries in sub-Saharan Africa [6]. However, disaggregated estimates of Se deficiency at sub-national scales are inadequate across sub-Saharan Africa, due to a lack of reliable spatial information on food systems or biomarkers of Se status [2].

Phiri et al. [8] recently reported a high prevalence of Se deficiency in Malawi, with 62.5% of women of reproductive age and 52.5% of adult men having plasma Se concentrations below a threshold of deficiency based on optimal GPx3 activity. However, consistent with previous small-scale cross-sectional studies [9], they found that Se status varied spatially, with a lower risk of deficiency in populations in southern Malawi and along Lake Malawi, and greater risk of deficiency in Central and Northern upland areas. This was attributed to potential differences in soil type and access to fish. Notably, the movement of Se from soils to crops, and hence, the entry of Se into the food system, is highly dependent on soil factors [10]. On some soil types (typically with high pH), Se can move readily into crops; on other soils (typically with low pH), Se is less available to plants. Households in Malawi typically consume locally-produced crops, and the observed sub-national variations in Se status were consistent with previous surveys of soil and crop Se status [11].

In Ethiopia, previous studies found that Se deficiency is a health concern in some areas. In a study of school-aged children (*n* = 555) in the Amhara Region, Gashu et al. [12] reported that 49% of children had Se inadequacy (using a serum Se concentration threshold of <70 μg L^−1^), but the prevalence of deficiency was much greater in Western than Eastern Amhara. In the city of Gondar, in North East Amhara Region, Se deficiency was reported among 30% of women of reproductive age (*n* = 26/87) [13] and 62% of school aged children (*n* = 62/100) [14]. Thus, Se deficiency is likely to be widespread in parts of Ethiopia, but geographically dependent, however, no data exist for the majority of the country.

The Ethiopian National Micronutrient Survey (ENMS) was conducted in 2015, with the aim of providing information on population micronutrient status at national and sub-national scales, to better inform policies and programs. A range of biomarkers of micronutrient status, including iodine, iron, zinc and vitamin A, but not Se, were measured in nationally- and regionally representative groups [15]. In the current study, Se concentration was measured in archived serum samples from the ENMS and geostatistical methods were applied to model the spatial variation of serum Se across the country. The results provide novel and comprehensive information on the prevalence and sub-national variation of population Se status in Ethiopia, contributing to the understanding of the public health significance of Se deficiency and informing potential interventions to alleviate Se deficiency.

## 2. Materials and Methods

### 2.1. Study Design and Population

**Figure 1:**
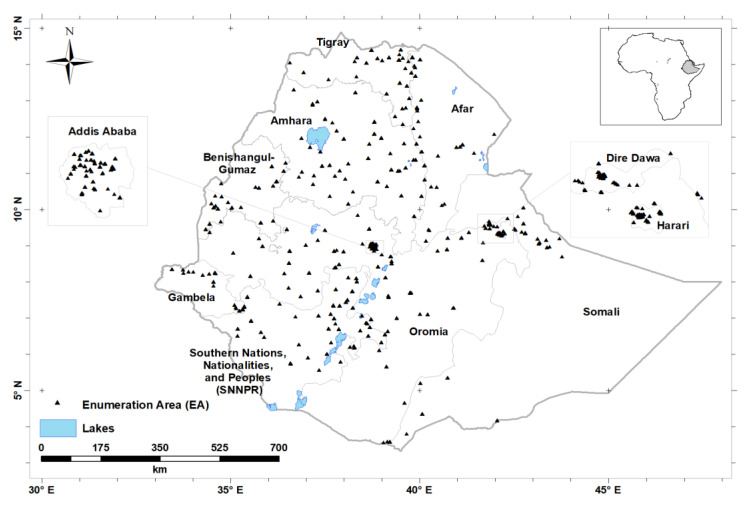
Locations of Enumeration Areas (*n* = 346), from which study participants were recruited.

The ENMS covered all nine regions of Ethiopia and two administrative cities (Addis Ababa and Dire Dawa; Figure 1). The design of the ENMS is explained in detail elsewhere [16]. Briefly, the ENMS was planned as a large, population-based, regionally and nationally-representative cross-sectional survey of young children (YC, aged 6–59 months, *n* = 1100), school-age children (SAC, aged 5–15 years, *n* = 1500), women of reproductive age (WRA, aged 15–49 years, *n* = 1600) and men (aged 15–54 years, *n* = 500), conducted between March and July 2015. The ENMS enumeration areas (EAs) or clusters are geographic areas defined by the Central Statistical Agency (CSA) for the Ethiopia Population and Housing Census [17]. The survey employed stratified sampling in each of the nine regions and two administrative cities. For each region, the EAs were selected based on standard probability proportional to size (PPS). Within each selected EA 11, households were randomly selected for enumeration. EAs contain on average 181 households (150 to 200) [17].

The present study used two datasets: socio-demographic data collected from georeferenced households in the ENMS, and newly generated data on Se concentration measured in archived serum samples from the ENMS. The data on serum Se concentration were obtained only for those individuals from the ENMS survey, for whom socio-demographic data and location information from GPS were available, and where there was a sufficient serum sample for analysis. A total of 3376 out of the 4700 archived serum samples contained sufficient material for analysis of Se concentration.

### 2.2. Data Collection and Analysis

#### 2.2.1. Socio-Demography

Socio-demographic information was collected in the ENMS through a structured questionnaire. The ENMS enumerators and supervisors were trained on data collection and quality control, and the questionnaire was pilot tested in a cluster that was not selected for the actual survey. Questionnaires were refined after the pilot testing and before the survey data collection began.

#### 2.2.2. Collection, Processing and Analysis of Serum Se

Blood collection and processing methods are described elsewhere [16]. Briefly, venous blood samples were collected from participants by trained phlebotomists and processed as per the World Health Organization (WHO) blood collection guidelines [18]. Samples were aliquoted in the field and subsequently analyzed for a range of micronutrients [16]. One aliquot of each sample was stored at EPHI at −80 °C as archived material, in case analyses needed to be repeated or additional micronutrient analyses could be supported, as is the case with the present study.

The concentration of Se in serum samples was determined using inductively coupled plasma mass spectrometry (ICPMS) at the University of Nottingham, UK (Thermo Fisher Scientific iCAPQ, Thermo Fisher Scientific, Bremen, Germany). Samples were introduced, via a single line, from an auto-sampler incorporating an ASXpress™ rapid uptake module (Cetac ASX-520, Teledyne Technologies Inc., Omaha, NE, USA), through a perfluoroalkoxy (PFA) microflow PFA-ST nebulizer (Thermo Fisher Scientific, Bremen, Germany). All samples and external element calibration standards were diluted as 0.3 mL added to 6 mL of a solution containing: (i) 0.5% HNO_3_ (Primar Plus grade); (ii) 2.0% methanol (Fisher Scientific UK Ltd., Loughborough, UK) and (iii) three internal standards including ^72^Ge (10 µg L^−1^), ^103^Rh (5 µg L^−1^), ^193^Ir (5 µg L^−1^) (SPEX Certiprep Inc., Metuchen, NJ, USA). Selenium calibration standards were prepared at 0, 20, 40, 100 µg L^−1^ (Claritas-PPT grade CLMS-2; SPEX Certiprep Inc., Metuchen, NJ, USA). The ICPMS was operated in ‘collision-reaction cell mode’, with kinetic energy discrimination, using H_2_ as the cell gas to maximize sensitivity for Se determination.

The limit of detection (LOD) for Se was measured as 3 times standard deviation of 10 operational blanks; the limit of quantification (LOQ) was calculated as 10 times standard deviation. The LOD and LOQ were 0.029 and 0.096 µg L^−1^, respectively. Accuracy was verified by the use of two Seronorm™ reference materials: L-1 (Lot 1801802) and L-2 (Lot 1801803) (Nycomed Pharma AS, Billingstad, Norway). These were prepared in an identical way to the samples and typically run at the start and the end of each analytical run. Average Se recovery (%; *n* = 24) when compared to accredited values determined across 10 analytical batches of blood serum was 93% and 95% for L-1 and L-2, respectively.

#### 2.2.3. Data Analysis

Of the total of 3376 serum samples with sufficient volume for re-analysis, 107 were excluded from the statistical analysis, due to missing GPS coordinates (*n* = 101; men = 5, YC = 63, SAC = 24 and WRA = 9), or a lack of demographic data (*n* = 5; 3 from SAC and 2 from WRA) and one outlier datum from WRA. Thus, 3269 observations were included to determine Se status of the study population. Thresholds for optimal activity of GPx3 (≥84.9 µg L^−1^) and IDI (≥64.8 µg L^−1^) in adults, used by Phiri et al. [8] and which were based on Thomson [19], were compared against serum Se concentration of WRA and men, to estimate the percentage of population at risk of oxidative stress and thyroid dysfunction as a result of Se deficiency, respectively. Data were also compared against a serum Se concentration threshold <70 µg L^−1^ [12]. Descriptive statistical analyses were conducted in STATA (Version 14.0, StataCorp LLP, College Station, Texas, USA). Survey weights were applied for summary statistics.

To map serum Se concentration at a national level, presented here for WRA only, ordinary kriging predictions were computed on the nodes of a 60-m square grid. This is a conditional distribution; conditional on the geostatistical model (the variogram in the case of ordinary kriging) and the observed values at sample locations. In ordinary kriging the prediction is the mean of the prediction distribution, and its variance is the ordinary kriging variance.

Data values were first aggregated to mean values for each EA, and mean coordinates were computed for each EA. One single value was a probable outlier according to the criteria of Tukey (1977) [20]. This value was removed for variogram estimation, but was returned for spatial prediction by kriging. This is because estimates of the variogram are particularly susceptible to extreme values [21], but if, as here, there is no reason to think the observation erroneous, then it should be considered for local prediction.

Because of the large extent of the sampled region, all spatial analyses were done using the latitude and longitude recorded for the observations, rather than on a rectilinear grid, since no single projection would be suitable at all locations. Under the usual assumptions of stationarity in geostatistics, spatial dependence is modelled in terms of the vector that separates locations, or lag. In this setting, the lag distance between any two points was computed as the great circle distance on a spherical approximation. This was computed using the *dist Vincenty Sphere* function from the geosphere package for the R platform [22]. The function *final Bearing* from the same package was used to obtain bearings. Note that, when distances between locations are measured on the sphere, not all variogram functions that are suitable for use on the plane can be applied. On the sphere the exponential model is authorized [23], and so it was used for all variogram modelling in this study. Our spatial analysis is focused on WRA, because thresholds linked to optimal activities of selenoproteins GPx3 and IDI are only established for adults [19], and because there were greater numbers of WRA in the sample. Statistical summary data for WRA are shown in Supplementary Information Figure S1.

Exploratory variogram analysis indicated that there was no marked directional dependence of the variogram (Supplementary Information/Figure S2), so an isotropic variogram (Supplementary Information/Figure S3) was estimated using the standard estimator due to Matheron (1962) [24]. In addition, variogram values were estimated using the robust estimators, due to Dowd (1984) [25] and to Cressie and Hawkins (1980) [26]. These alternative estimators were considered because, although a marginal outlier (an unusual datum as it appears on the overall distribution) was removed; spatial outliers (observations unusual in their spatial context) can inflate estimates of the semivariance [21]. An exponential variogram model [27] was fitted by weighted least squares, and then tested by cross-validation. In cross-validation each observation was, in turn, removed and predicted from the remaining observations by ordinary kriging. The squared cross-validation error at each site was standardized by dividing by the ordinary kriging variance. The median standardized squared prediction error (SSPE) has an expected value of 0.455 in the case of a valid underlying variogram model with kriging errors that appear to be normally distributed [20]. The estimator due to Matheron (1962) [24] is more statistically efficient than the robust alternatives, so if the model fitted to these estimates appeared to be correct from the cross-validation results (median SSPE) then the alternatives were not considered. If the SSPE suggests that the model fitted to Matheron estimates are affected by outliers, then the models fitted to robust estimates are also cross-validated, and one is selected on the cross-validation results. This procedure follows the recommendations of Lark [21].

### 2.3. Ethical Approval

Ethical approval for the ENMS was obtained from the National Research Ethical Review Committee of the Ethiopian Science and Technology Ministry (Reference 3.10/433/06). Informed consent was obtained from all adult participants and assent for all child participants in the survey. A separate ethical approval was obtained for the present study from the Research Ethical Review Committee of the Ethiopian Public Health Institute (Protocol EPHI-IRB-140-2018). Archived serum samples were transferred from storage at EPHI to the University of Nottingham, UK, for analysis under a material transfer agreement.

## 3. Results

### Characteristics of Study Participants

The largest number of participants was from Oromia Region and the smallest number from Dire Dawa administrative city area (Table 1). Among 3269 observations, 15.9% were YC, 30.8% were SAC, 12.8% were Men and 40.6% were WRA. About a quarter of the participants were from urban areas. Around half of SAC (*n* = 451/1007) and YC (*n* = 284/521) were male (Table 1).

**Figure 2:**
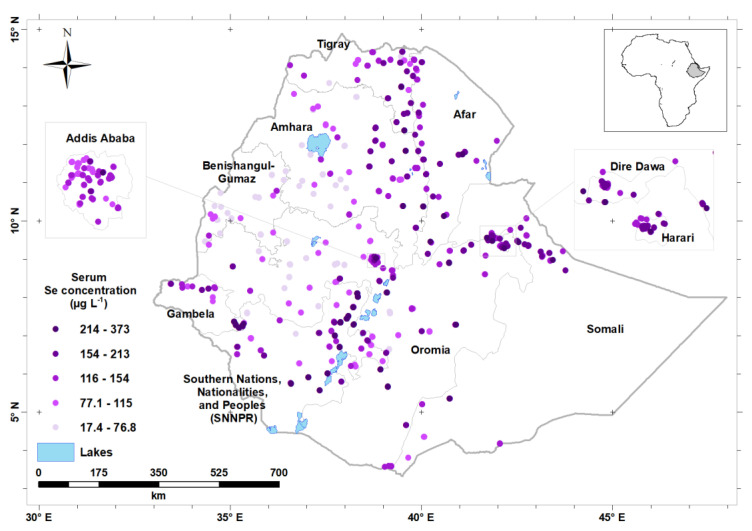
Distribution of mean serum selenium (Se) concentration (by enumeration area) in Ethiopia.

The median serum Se concentration of the study population was 87.7 μg L^−1^ and the overall prevalence of Se deficiency was 35.5%. Serum Se concentration was highly variable by region, with a median (Q1, Q3) of 54.6 (43.1, 66.3) μg L^−1^ in Benishangul-Gumuz Region and 122.0 (105.0, 142.0) μg L^−1^ in Afar Region, and 122.0 (85.3, 171.0) in the Southern Nations, Nationalities, and Peoples’ Region (SNNPR) (Table 2). The lowest prevalence of Se deficiency (<5%) was observed in North-East and Eastern parts of the country, which includes Dire Dawa, Somali, and Afar Regions. In contrast, the highest prevalence of Se deficiency was observed in North-West and Western part of the country, including Benishangul-Gumuz (87.2%), Amhara (54.9%) and Oromia (43.7%) Regions (Table 2). Figure 2 shows serum Se concentration by enumeration area.

Median serum Se concentrations varied by demographic group, being greatest among WRA 103.6 (68.2, 143.0) µg L^−1^ and smallest among YC 67.2 (41.9, 94.7) µg L^−1^ (Table 2). Different thresholds of serum Se concentration have been used to define Se deficiency, based on associated expression of Se-containing proteins [8,19]. Serum Se concentration was below a threshold for optimal GPx3 activity [8] in 35.4% of WRA and 42.9% of men. In addition, serum Se concentration was below a threshold for optimal IDI activity [8] in 22.4% of WRA and 28.8% of men. Compared to a threshold of 70 µg L^−1^, as used previously in Ethiopia [5,12], 35.5% of the sample population were deficient (Table 2).

**Figure 3:**
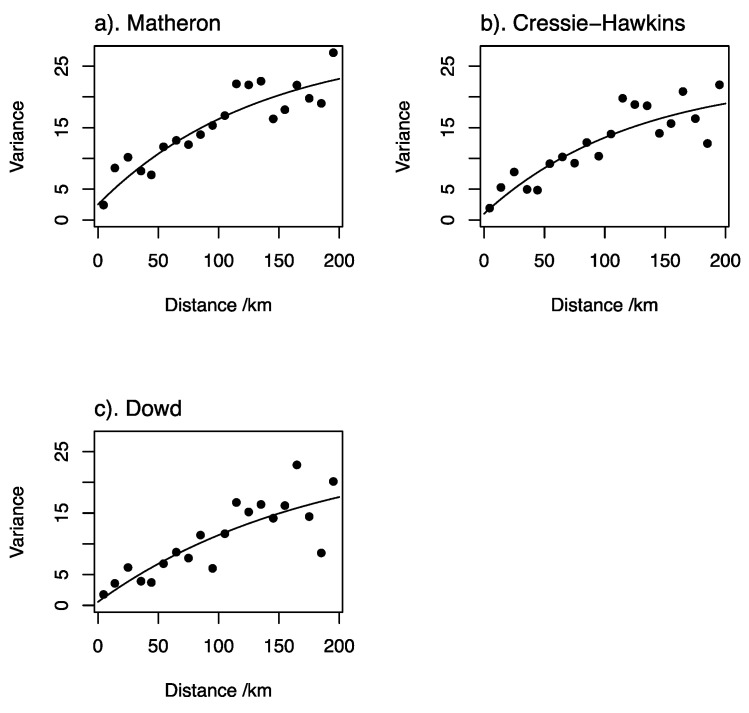
Variograms of serum Se concentration in women of reproductive age (WRA) in Ethiopia estimated by different estimators; (**a**) Matheron, (**b**) Cressie-Hawkins and (**c**) Dowd.

**Figure 4:**
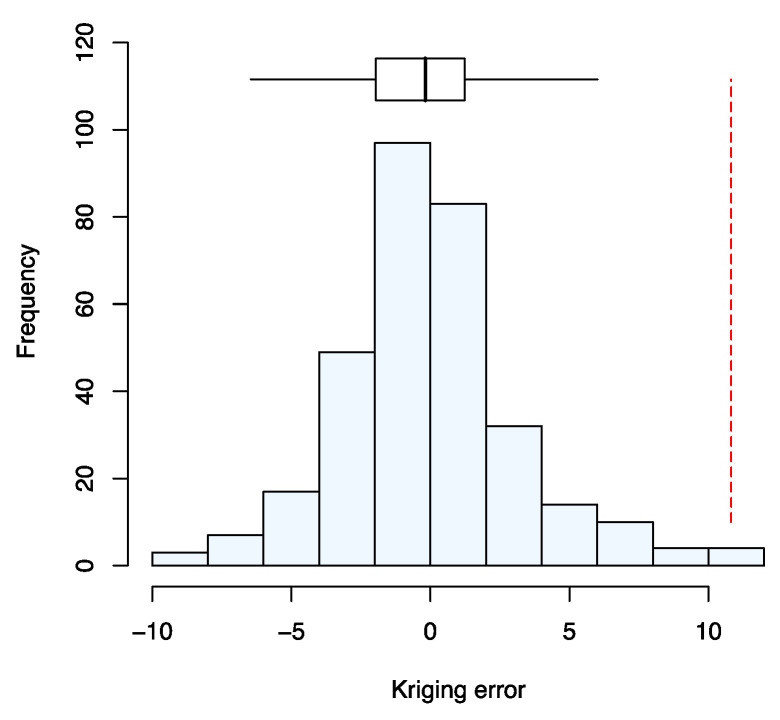
Histogram of the cross-validation errors using the variogram fitted to estimates obtained with the Matheron estimator [24]. The Box-plot shows the median and quartiles (box limits). The vertical dashed line shows the outer fence values of Tukey [20].

Figure 3 shows the estimates of the variogram for serum Se concentration by the three estimators, with exponential models fitted. The cross-validation errors for the model fitted to the estimates obtained following Matheron [24] appeared to be normally distributed (Figure 4). The median SSPE was 0.41; this falls within the 95% confidence interval for the value expected when the model is valid (0.34–0.57) and is close to the expected value (0.455). The parameters of the cross-validated exponential model are nugget variance (variation uncorrelated at scales resolved by spatial sampling 2.51; spatially correlated variance 26.23; distance parameter 132.8 km).

There is substantial spatial dependence in serum Se concentration in Ethiopia. Only a small proportion of the overall variance is attributable to a spatially uncorrelated nugget effect (which includes measurement error), and the correlated variance is spatially correlated up to distances of 200 km, as seen in the variograms in Figure 3 [28]. This implies that the variable under study is determined by factors that vary over large distances. This could be due to type and properties of soils, or wider landscape and climatic variation [29].

**Figure 5:**
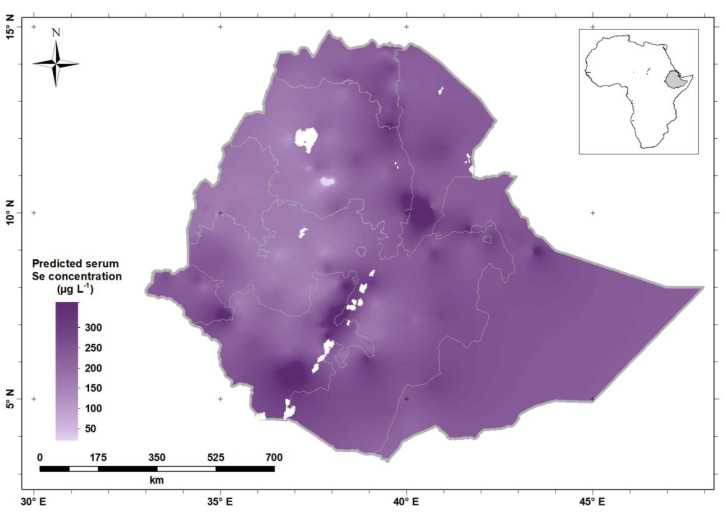
Predicted serum Se concentration (the mean of the prediction distribution) in women of reproductive age in Ethiopia.

The greatest serum Se concentrations (darker shade of purple) were observed along the Rift Valley of Ethiopia and in the North-East and Eastern parts of Ethiopia, including Afar, Somali and Harari Regions, and Dire Dawa City. Selenium status is low (lighter shade of purple) in the North-western and Western parts of Ethiopia, including Benshangul-Gumuz Region and large parts of Amhara and Oromia Regions, including in highland areas either side of the Rift Valley. Figure 5 shows the predicted serum Se concentration for WRA across Ethiopia, obtained by ordinary kriging from the data.

**Figure 6:**
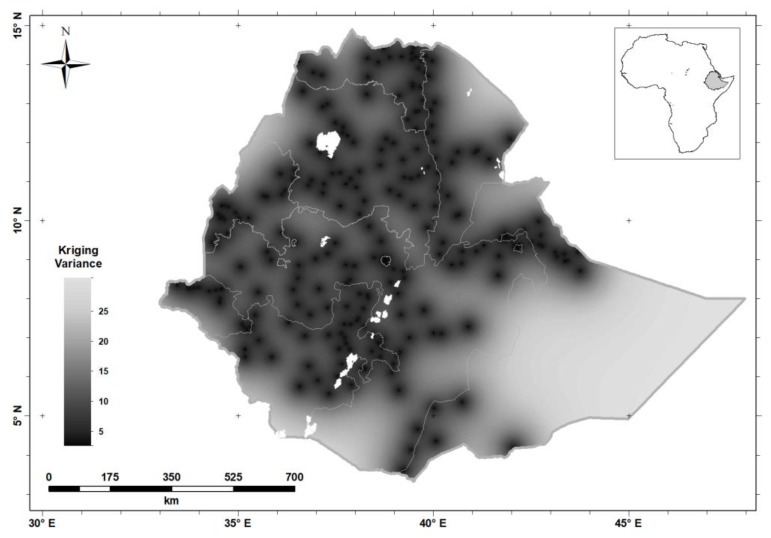
Serum Se concentration kriging variance (the variance of the prediction distribution) in women of reproductive age in Ethiopia.

Figure 6 shows the kriging variances of the predictions of serum Se concentration for WRA; these are the expected squared prediction errors computed on the basis of the variogram model. The kriging variance (Figure 6) is the measure of prediction uncertainty that allows one to visualize uncertainty made from sparse observations.

Interpolation error is inevitable because of the spatial variation of our target variable, and the fact that predictions are made from sparse observations. The kriging variance (Figure 6) allows us to visualize this uncertainty. In particular, it is apparent that there is greater uncertainty in the Eastern part of the country, because of the very sparse distribution of observations [30]. In these circumstances the predicted value tends to the mean of the observations, and the kriging variance is large. If decisions must be made in regions where the kriging variance is large, then local sampling would be essential. However, this might be true of locations in the West of the country for a variable which shows spatial dependence only over very short distances (e.g., tens of kilometers). Examining the kriging variance allows us to avoid arbitrary decisions as to where spatial prediction is reliable from a particular distribution of observations. We consider the implications of prediction uncertainty further below, but first we examine the general spatial variation revealed by the kriging predictions.

**Figure 7:**
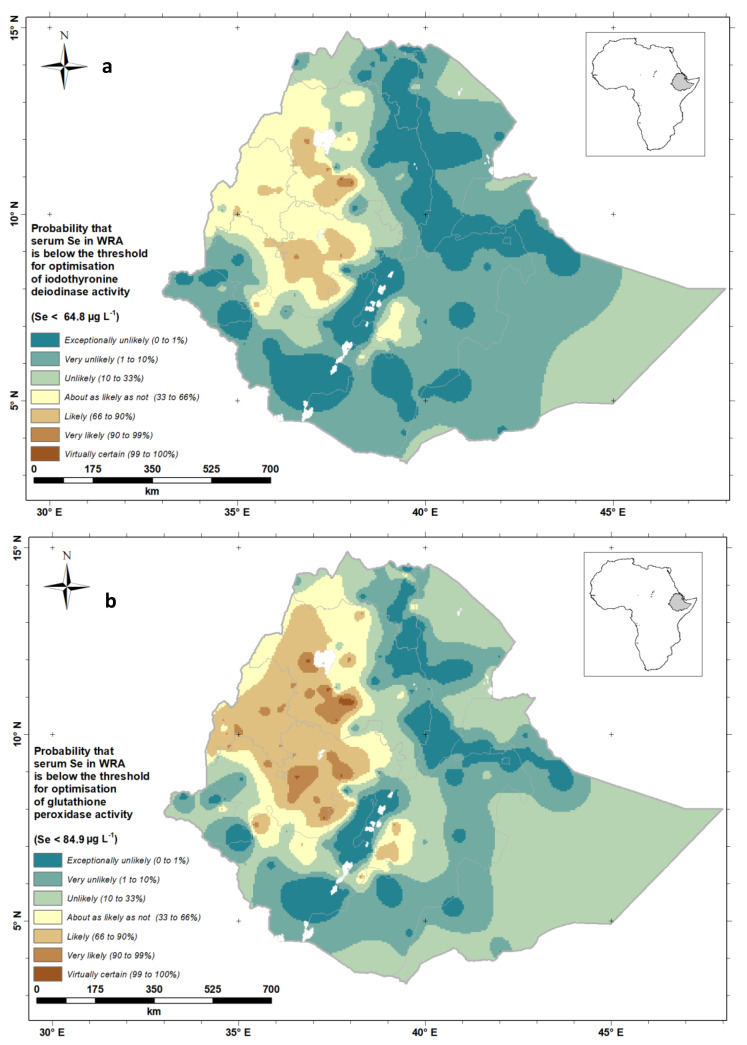
Probability that serum Se concentration of Ethiopian women of reproductive age would fall below a threshold for the optimal activity of (**a**) iodothyronine deiodinase (IDI), (**b**) glutathione peroxidase 3 (GPx3).

The output from the geostatistical analysis allows us to visualize the probability of Se deficiency on a national scale. Figure 7 shows the probability that the mean serum Se concentration of WRA in an EA at a location is below a threshold for the optimal activities of IDI (Figure 7a) and GPx3 (Figure 7b). Because continuous probabilities are not always easily interpreted by diverse stakeholders, we also present these probabilities using the “calibrated phrases” of the Intergovernmental Panel for Climate Change (IPCC) [31], which are designed for the communication of uncertain information to data users unfamiliar with probability. This approach has been used elsewhere [8,32].

From the probability maps (Figure 7a,b), we can see that WRA in large parts of Western Ethiopia are ‘likely’ through to ‘virtually certain’ to have deficient Se status, leading to impaired GPx3 and IDI activity, with implications for antioxidant and thyroid functions, respectively. Deficiency is also ‘likely’ or ‘very likely’ in the Bale Zone to the east of the Rift Valley. Along the Rift Valley and in large parts of Tigray (North-East Ethiopia), Eastern and Southern Ethiopia, WRA are ‘unlikely’ through to ‘exceptionally unlikely’ to be Se deficient.

## 4. Discussion

This study reports the first national-scale estimates of Se deficiency in Ethiopia, finding that the national median (Q1, Q3) serum Se concentration was 87.7 (56.7, 123.0) μg L^−1^. Slightly over one third of the sample population were Se deficient. However, the prevalence of deficiency was highly variable, with almost no deficiency in Afar (0.9%), Somali (3.5%) and Dire Dawa (2.8%), whereas the majority of the population (81.2%) were deficient in Benishangul-Gumuz. Serum Se concentration showed marked variation between demographic groups (e.g., YC and SAC had lower serum Se concentrations than adult men and WRA).

The majority of between-individual variation in the sample population is attributable to location of residency. Selenium status was substantially higher (and likelihood of deficiency much lower) along the Rift Valley, extending from the Afar Triple Junction at the Red Sea Gulf of Aden intersection to the Kenyan Rift, in the dry Tigray and Somali Regions, and in lowland areas of Gambella Region. Selenium status was low (and prevalence of deficiency high) in upland areas of Amhara, Oromia and Benishangul-Gumuz Regions, and in the elevated Bale Zone. The spatial factors influencing Se status operated at distances up to 200 km, likely driven by factors including geological determinants of soil properties, climate and landscape.

Selenium status of populations in other areas of the globe varies from as low as <50 μg L^−1^ in some parts of China and Serbia, to high concentrations (>200 μg L^−1^), in some districts of USA and other parts of China. However, the majority of population average serum Se concentrations fall within the range 80–120 μg L^−1^ [33], and the median serum Se concentration in the present study (87.7 μg L^−1^) lies towards the lower end of this range. In Ethiopia, the national prevalence of Se deficiency was previously estimated based on Se supplies in the food system, estimating that 36% of the population are at risk of deficiency due to inadequate dietary Se supplies [6]. A high prevalence of Se deficiency has been reported previously among YC in Western Amhara, but little or no prevalence in populations from Eastern Amhara [12,34]. This is consistent with the present study, which also finds a strong gradient of Se status across the Amhara Region.

Several factors may contribute to variations in serum Se concentrations. These include short-term physiological factors including presence of infection, and long-term influences including dietary patterns, age and sex [35,36]. However, in Ethiopia, spatial factors linked to soil types and landscape features appear to be strong drivers of longer-range variation in Se status [29]. Food systems are highly localized in Ethiopia, particularly in rural areas, with a large proportion of dietary intakes met through subsistence production or purchases of locally-produced food [37]. Thus, individuals’ status reflects the soil types and landscapes where they reside.

Ethiopia is located towards the Northern end of the East African Rift Valley. Within the Rift Valley, there are Holocene and active volcanoes which have discharged volcanic ash, lava and gases contributing to the formation of fertile volcanic soils, including with high concentrations of plant-available Se [38]. There are different pathways for volcanic activity to impact Se status: intake by drinking water and food. Drinking water and food are considered the most vital sources of trace elements for humans [39]. Another important geographical feature affecting Se status may be the Rift Valley lakes, which in Ethiopia include the Abaya, Chamo, Zeway, Shala, Koka, Langano, Abijatta and Hawassa Lakes. It is likely that the consumption of fish—which is a good dietary source of Se—is greater among groups living near to these lakes, as was noted previously in Malawi [8].

The present study establishes a baseline of population Se status in Ethiopia, which could inform programs or policies to alleviate Se deficiency, as well as future surveillance programs. Potential strategies to alleviate Se deficiency include promotion of dietary diversity and agronomic biofortification, i.e., the application of Se to crops via fertilizers [40,41]. Geographical targeting of these interventions would appear to be warranted, while denser sampling is clearly required in parts of the country (notably Eastern Ethiopia) before information is adequate to guide any interventions. Further research is also required to determine the health significance of the deficiencies observed in the current study.

Selenium plays its biological role mediated through several selenoproteins, including glutathione peroxidases (GPx), iodothyronine deiodinases (IDI), Selenoprotein P, etc. We defined Se deficiency using a threshold serum Se concentration <70 µg L^−1^, as used previously, including for children in Ethiopia [5,12,34]. In the geostatistical analysis in this study, we used thresholds based on the optimal expression of two selenoproteins, GPx3 and IDI, based on Thomson [19]. Thus, the GPx3 threshold was the mid-point between 1.00 and 1.15 µmol Se/L, as used by Phiri et al. (2019) [8]. Due to differences between demographic groups in the amount of Se needed for biosynthesis and the expression of selenoproteins, it may be appropriate to apply different thresholds of deficiency to specific demographic groups [42,43]. Furthermore, higher thresholds of concentration may be appropriate among adults, given the potential protective effect of Se against cancer observed in the USA [44] and Europe [45]. However, until further evidence is available to support the determination of age- and sex-specific cut-offs, there is a need to interpret reported prevalence of deficiency with caution—it may be substantially greater. However, it should also be noted that Se is a negative acute phase reactant, meaning that its concentration in serum is suppressed during inflammation [46], so applications of thresholds derived from Western populations may not be appropriate.

The strengths of our study include a large population coverage of multiple demographic groups based on nationally representative sampling in a cross-sectional design. We used accurate instrumental analyses and advanced geostatistical methods to predict the Se status of a population and elucidate the area where the probability of GPx3 and IDI activities are low due to Se deficiency. The study limitations include a lack of analyses of potential contributors to serum Se concentration in different areas; for example, data on soil, crop, and livestock Se status, which could now be used to determine the causal factors which are responsible for serum Se concentration.

## 5. Conclusions

The present study reveals that Se deficiency in Ethiopia is widespread. The risk of Se deficiency is highly spatially dependent, which is likely to be due to environmental factors including soil type and landscape features, and the localized nature of food production and distribution [8,29,47]. These findings suggest the need to investigate strategies that can increase dietary intake of Se in deficient areas. In addition, the strong spatial variation in Se status suggests that policies to alleviate deficiency should probably be spatially targeted for efficiency and to ensure the right people are reached. Agronomic biofortification of staple crops with Se may be a cost-effective way to alleviate Se deficiency in populations [48]. Selenium-containing fertilizers could be targeted to areas of Ethiopia with a high prevalence of Se deficiency. In addition, increased dietary diversification and the promotion of crops that naturally accumulate high concentrations of Se, such as the drumstick tree (*Moringa oleifera* and *Moringa stenopetala*), could contribute to greater Se intakes [49].

## Figures and Tables

**Table 1:** Characteristics of participants in the Ethiopian National Micronutrient Survey, 2015.

Characteristics			n	Percentage
Region		Addis Ababa	243	7.4
		Afar	254	7.8
		Amhara	492	15.1
		Benishangul-Gumuz	213	6.5
		Dire Dawa	152	4.7
		Gambela	199	6.1
		Harari	243	7.4
		Oromia	523	16.0
		SNNP	365	11.1
		Somali	204	6.3
		Tigray	381	11.6
		National	3269	100.0
Demographic group	Young children	Male	284	15.9
		Female	237	
	School age children			
		Male	451	30.8
		Female	556	
	Adult Men		414	12.7
	Women of reproductive age		1327	40.6
Residence	Urban		841	25.7
	Rural		2428	74.3
Gender	Male		1149	35.1
	Female		2120	64.9

**Table 2:** Serum Se concentrations and prevalence of deficiency by region and demographic group in the Ethiopian National Micronutrient Survey, 2015.

Variables		n	Median Serum Se (µg L^−1^) ^†^	Prevalence (%) of Se Deficiency (Serum Se <70 µg L^−1^)
Region	Addis Ababa	243	92.7 [80.2, 111.0]	7.1
	Afar	254	122.0 [105.0, 142.0]	0.9
	Amhara	492	67.4 [44.7, 94.7]	52.2
	Benishangul-Gumuz	213	54.6 [43.1, 66.3]	81.2
	Dire Dawa	152	118.9 [106.0,137.0]	2.8
	Gambela	199	114.8 [88.5,157.0]	16.1
	Harari	243	92.2 [62.9,114.0]	32.8
	Oromia	523	76.8 [48.8,114.0]	44.4
	SNNP	365	122.0 [85.3,171.0]	14.1
	Somali	204	118.0 [100,141.0]	3.5
	Tigray	381	104.0 [82.3, 128.0]	15.5
	National	3269	87.7 [56.7, 123.0]	35.5
Demographic group	Young children	521	67.2 [41.9, 94.7]	52.3
	School age children	1007	78.7 [53.4,107.8]	40.6
	Men	414	94.8 [60.4, 128.0]	30.8
	WRA	1327	103.6 [68.2,143.0]	26.0

SNNP: Southern Nations, Nationalities, and Peoples’; WRA: women of reproductive age, ^†^ values in brackets are Q1: 25th percentile and Q3: 75th percentile.

## Supplementary Materials

The following are available online at https://www.mdpi.com/2072-6643/12/6/1565/s1, Figure S1: Summary statistics for WRA, Figure S2: Directional-dependent estimates of the variogram, Figure S3: Estimates of the isotropic variogram.

## References

[1] Jones G.D., Droz B., Greve P., Gottschalk P., Poffet D., McGrath S.P., Senevirante S.I., Smith P., Winkel L.H.E. (2017). Selenium deficiency risk predicted to increase under future climate change. Proc. Natl. Acad. Sci. USA.

[2] Rayman M.P. (2012). Selenium and human health. Lancet.

[3] Brown K.M., Arthur J.R. (2001). Selenium, selenoproteins and human health: A review. Public Health Nutr..

[4] Fairweather-Tait S.J., Bao Y.P., Broadley M.R., Collings R., Ford D., Hesketh J.E., Hurst R. (2011). Selenium in human health and disease. Antioxid. Redox Signal..

[5] Gashu D., Stocker B.J., Preedy V., Patel V. (2017). Selenium and Cognition: Mechanism and Evidence. Handbook of Famine, Starvation, and Nutrient Deprivation.

[6] Joy E.J.M., Ander E.L., Young S.D., Black C.R., Watts M.J., Chilimba A.D.C., Chilima B., Siyame E.W.P., Kalimbira A.A., Hurst R. (2014). Dietary mineral supplies in Africa. Physiol. Plant..

[7] Ligowe I.S., Phiri F.P., Ander E.L., Bailey E.H., Chilimba A.D.C., Gashu D., Joy E.J.M., Lark R.M., Kabambe V., Kalimbira A.A. (2020). Selenium (Se) deficiency risks in sub-Saharan African food systems and their geospatial linkages. Proc. Nutr. Soc..

[8] Phiri F.P., Ander E.L., Bailey E.H., Chilima B., Chilimba A.D.C., Joy E.J.M., Kalimbira A.A., Kumssa D.B., Lark R.M., Phuka J.C. (2019). The risk of selenium deficiency in Malawi is large and varies over multiple spatial scales. Sci. Rep..

[9] Hurst R., Siyame E.W.P., Young S.D., Chilimba A.D.C., Joy E.J.M., Black C.R., Ander E.L., Watts M.J., Chilima B., Gondwe J. (2013). Soil-type influences human selenium status and underlies widespread selenium deficiency risks in Malawi. Sci. Rep..

[10] Fordyce F. (2007). Selenium geochemistry and health. AMBIO.

[11] Chilimba A.D.C., Young S.D., Black C.R., Rogerson K.B., Ander E.L., Watts M.J., Lammel J., Broadley M.R. (2011). Maize grain and soil surveys revael suboptimal dietrary selenium intake is widespread in Malawi. Sci. Rep..

[12] Gashu D., Stoecker B.J., Adish A., Haki G.D., Bougma K., Aboud F.E., Marquis G.S. (2016). Association of serum selenium with thyroxin in severely iodine-deficient young children from the Amhara region of Ethiopia. Eur. J. Clin. Nutr..

[13] Tekeste Z., Amare B., Asfaw F., Fantahun B., van Nguyen N.V., Nishikawa T., Yabutani T., Okayasu T., Ota F., Kassu A. (2015). Determination of trace elements in Ethiopian, Vietnamese, and Japanese women using high-resolution IC-PMS. Nutrition.

[14] Amare B., Moges B., Fantahun B., Tafess K., Woldeyohannes D., Yismaw G., Ayane T., Yabutani T., Mulu A., Ota F. (2012). Micronutrient levels and nutritional status of school children living in Northwest Ethiopia. Nutr. J..

[15] UNICEF, World Bank, World Food Program, USAID ENGIN (2016). Ethiopian National Micronutrient Survey Report. https://www.ephi.gov.et/images/pictures/download2009/National_MNS_report.pdf.

[16] Tessema M., De Groote H., Brouwer I.D., Feskens E.J.M., Belachew T., Zerfu D., Belay A., Demelash Y., Gunaratna N.S. (2019). Soil zinc is associated with serum zinc but not with linear growth of children in Ethiopia. Nutrients.

[17] Population Census Commission (2008). Summary and Statistical Report of the 2007 Population and Housing Census. Population Size by Age and Sex.

[18] WHO (2010). WHO Guidelines on Drawing Blood: Best Practices in Phlebotomy.

[19] Thomson C.D. (2004). Assessment of requirements for selenium and adequacy of selenium status: A review. Eur. J. Clin. Nutr..

[20] Tukey J.W. (1977). Exploratory Data Analysis.

[21] Lark R.M. (2000). A comparison of some robust estimators of the variogram for use in soil survey. Eur. J. Soil Sci..

[22] Hijmans R.J. (2017). Geosphere: Spherical Trigonometry. R Package Version 1.5-7. https://CRAN.R-project.org/package=geosphere.

[23] Gneiting T. (2013). Strictly and non-strictly positive definite functions on spheres. Bernoulli.

[24] Matheron G. (1962). Traité de Géostatistique Appliqué, Tome 1. Memoires du Bureau de Recherches Géologiques et Minières.

[25] Dowd P.A., Verly G., David M., Journel A.G., Marechal A. (1984). The variogram and kriging: Robust and resistant estimators. Geostatistics for Natural Resources Characterization.

[26] Cressie N., Hawkins D.M. (1980). Robust estimation of the variogram. Math. Geol..

[27] Oliver M.A., Webster R. (2015). Basic Steps in Geostatistics: The Variogram and Kriging.

[28] Tadayon V., Torabi M. (2018). Spatial models for non-Gaussian data with covariate measurement error. Environmetrics.

[29] Gashu D., Lark R.M., Milne A.E., Amede T., Bailey E.H., Chagumaira C., Dunham S.J., Gameda S., Kumssa D.B., Mossa A.W. (2020). Spatial prediction of the concentration of selenium (Se) in grain across part of Amhara Region, Ethiopia. Sci. Total Environ..

[30] Goovaerts P. (1997). Geostatistics for Natural Resource Evaluation.

[31] Mastrandrea M.D., Field C.B., Stocker T.F., Edenhofer O., Ebi K.L., Frame D.J., Held H., Kriegler E., Mach K.J., Matschoss P.R. (2010). Guidance Note for Lead Authors of the IPCC Fifth Assessment Report on Consistent Treatment of Uncertainties.

[32] Lark R.M., Ander E.L., Cave M.R., Knights K.V., Glennon M.M., Scanlon R.P. (2014). Mapping trace element deficiency by cokriging from regional geochemical soil data: A case study on cobalt for grazing sheep in Ireland. Geoderma.

[33] Stoffaneller R., Morse N.L. (2015). A review of dietary selenium intake and selenium status in Europe and the Middle East. Nutrients.

[34] Gashu D., Marquis G.S., Bougma K., Stoecker B.J. (2019). Spatial variation of human selenium in Ethiopia. Biol. Trace Elem. Res..

[35] Maehira F., Luyo G.A., Miyagi I., Oshiro M., Yamane N., Kuba M., Nakazato Y. (2002). Alterations of serum selenium concentrations in the acute phase of pathological conditions. Clin. Chim. Acta..

[36] Galan P., Viteri F.E., Bertrais S., Czernichow S., Faure H., Arnaud J., Chenal S., Arnault N., Favier A., Roussel A.M. (2005). Serum concentrations of β-carotene, vitamins C and E, zinc and selenium are influenced by sex, age, diet, smoking status, alcohol consumption and corpulence in a general French adult population. Eur. J. Clin. Nutr..

[37] Central Statistical Agency of Ethiopia (2012). Household Consumption and Expenditure Survey 2010/11: Analytical Report. Statistical Bulletin 563.

[38] Floor G.H., Román-Ross G. (2012). Selenium in volcanic environments: A review. Appl. Geochem..

[39] Hartikainen H. (2005). Biogeochemistry of selenium and its impact on food chain quality and human health. J. Trace Elem. Med. Biol..

[40] White P.J., Broadley M.R. (2009). Biofortification of crops with seven mineral elements often lacking in human diets—Iron, zinc, copper, calcium, magnesium, selenium and iodine. New Phytol..

[41] Chilimba A.D.C., Young S.D., Black C.R., Meacham M.C., Lammel J., Broadley M.R. (2012). Agronomic biofortification of maize with selenium (Se) in Malawi. Field Crop. Res..

[42] Schomburg L., Schweizer U. (2009). Hierarchical regulation of selenoprotein expression and sex-specific effects of selenium. Biochim. Biophys. Acta.

[43] Kafai M.R., Ganji V. (2003). Sex, age, geographical location, smoking, and alcohol consumption influence serum selenium concentrations in the USA: Third National Health and Nutrition Examination Survey, 1988–1994. J. Trace Elem. Med. Bio..

[44] Bleys J., Navas-Acien A., Guallar E. (2008). Serum selenium levels and all-cause, cancer, and cardiovascular mortality among US adults. Arch. Intern. Med..

[45] Muecke R., Schomburg L., Buentzel J., Kisters K., Micke O., German Working Group Trace Elements and Electrolytes in Oncology-AKTE (2010). Selenium or no selenium-that is the question in tumor patients: A new controversy. Integr. Cancer Ther..

[46] Stefanowicz F.A., Talwar D., O’Reilly D.S., Dickinson N., Atkinson J., Hursthouse A.S., Rankin J., Duncan A. (2013). Erythrocyte selenium concentration as a marker of selenium status. Clin. Nutr..

[47] Joy E.J.M., Kumssa D.B., Broadley M.R., Watts M.J., Young S.D., Chilimba A.D.C., Ander E.L. (2015). Dietary mineral supplies in Malawi: Spatial and socioeconomic assessment. BMC Nutr..

[48] Alfthan G., Eurola M., Ekholm P., Venäläinen E.-R., Root T., Korkalainen K., Hartikainen H., Salminen P., Hietaniemi V., Aspila P. (2015). Effects of nationwide addition of selenium to fertilizers on foods, and animal and human health in Finland: From deficiency to optimal selenium status of the population. J. Trace Elem. Med. Biol..

[49] Kumssa D.B., Joy E.J.M., Young S.D., Odee D.W., Ander E.L., Broadley M.R. (2017). Variation in the mineral element concentration of *Moringa oleifera* Lam. and *M. stenopetala* (Bak. f.) Cuf.: Role in human nutrition. PLoS ONE.

