# Supplementary material for: Selenium Deficiency Is Widespread and Spatially Dependent in Ethiopia
Source: Nutrients. 2020 May 27;12(6):1565. doi: 10.3390/nu12061565 (PMC7353016; doi:10.3390/nu12061565)

Figure S1: Summary statistics for WRA

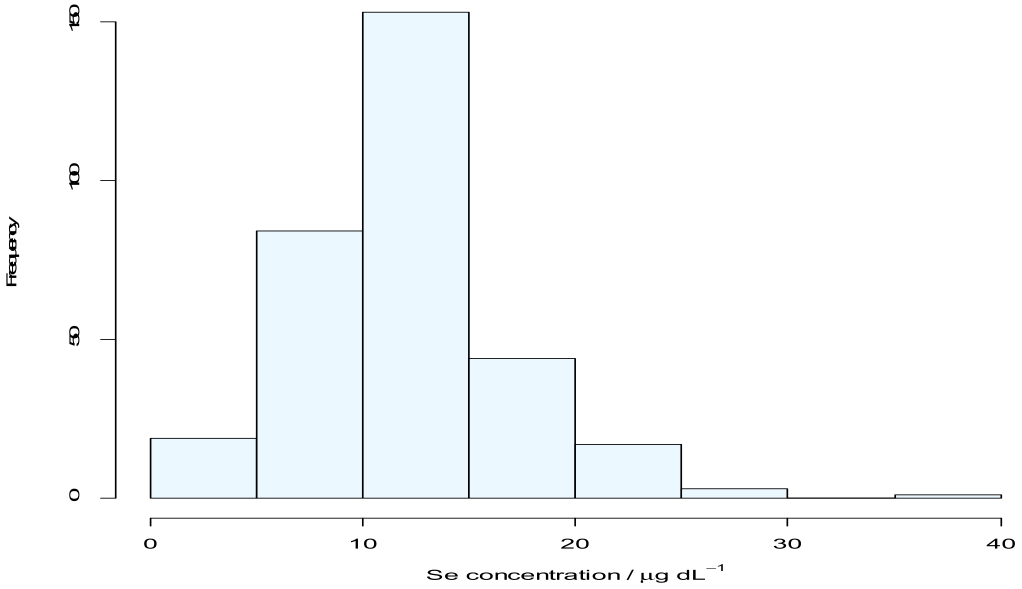

Figure S2: Directional-dependent estimates of the variogram

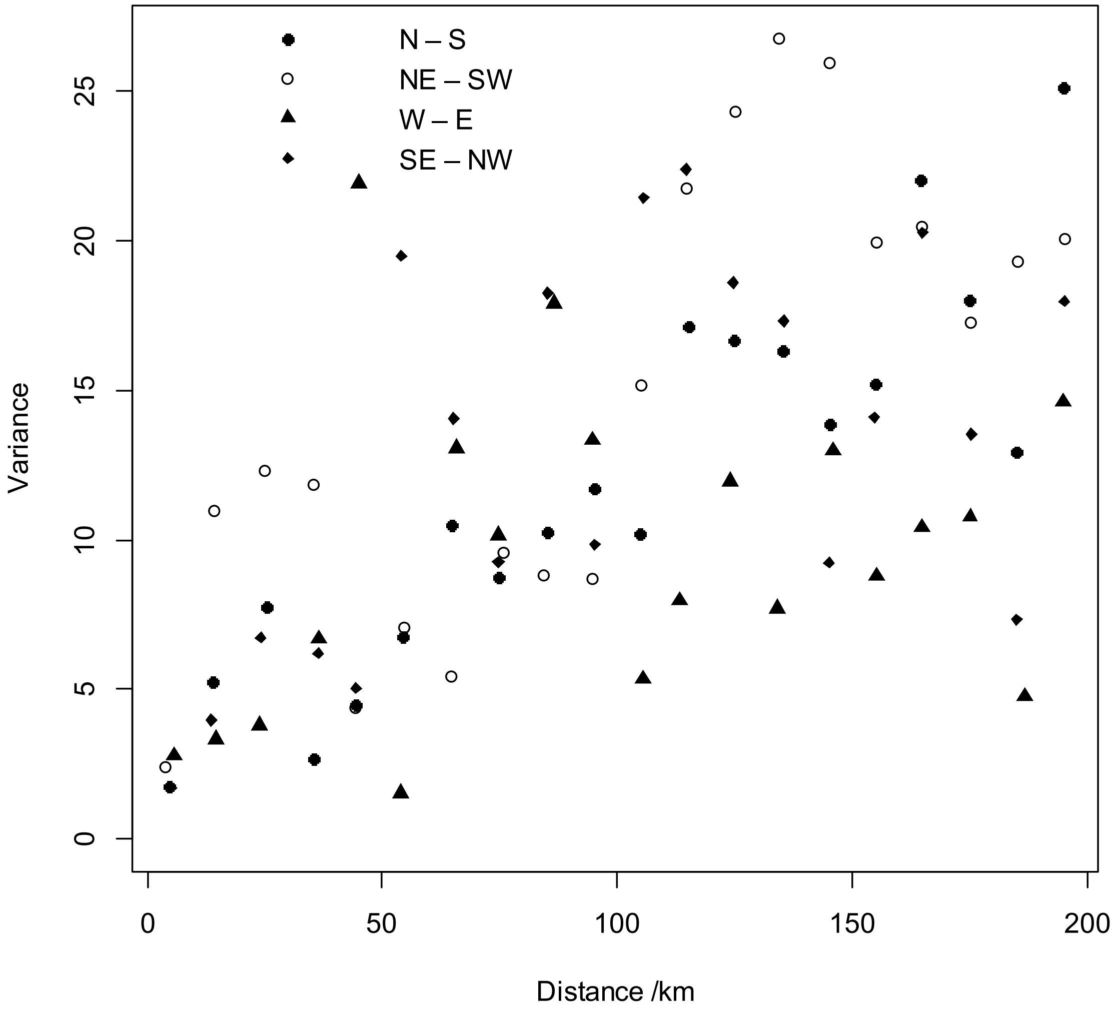

Figure S3: Estimates of the isotropic variogram

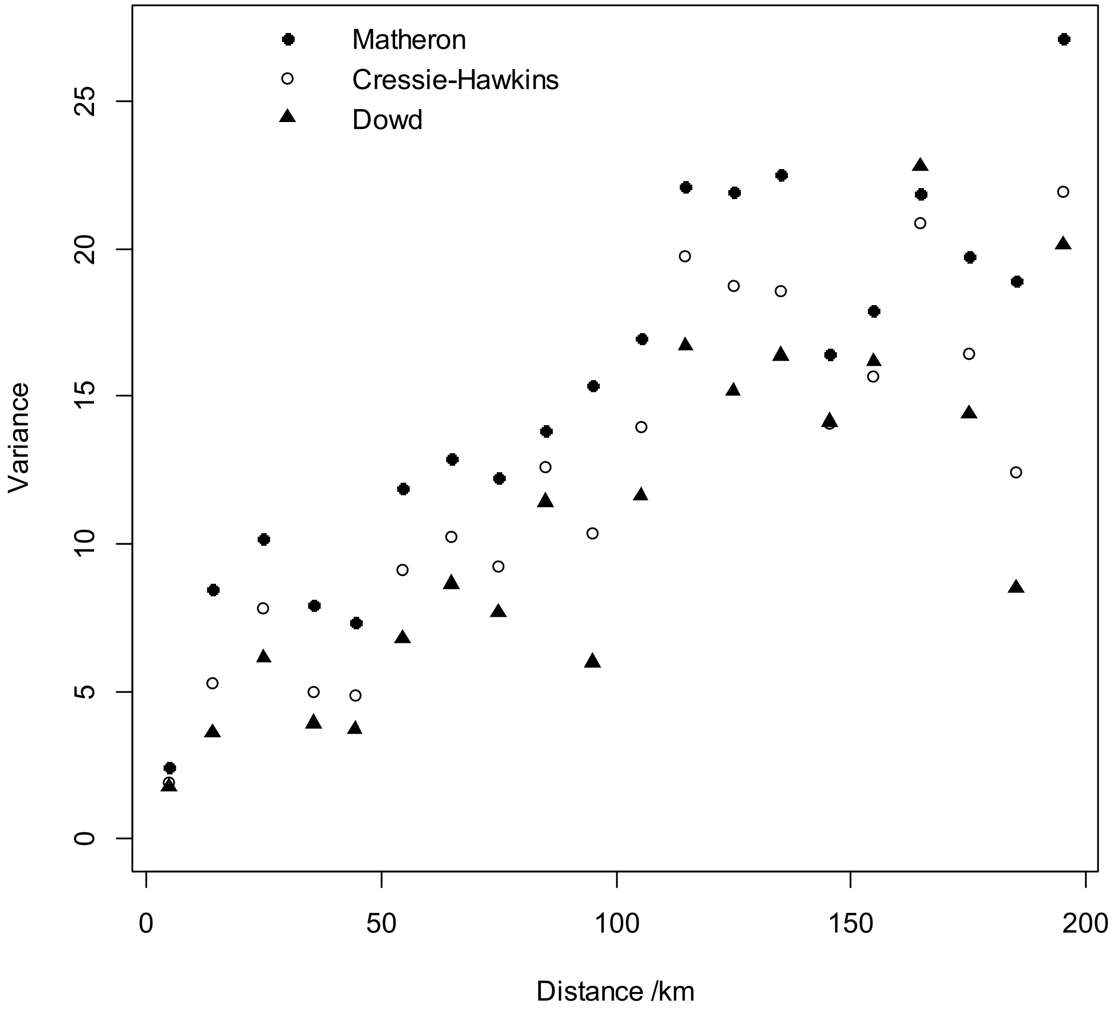

Supplement: Supplementary file 1 [file nutrients-12-01565-s001.pdf]
